# Effectiveness and tolerability of cephalexin and clavulanic acid fixed dose combination, amoxicillin and clavulanic acid fixed dose combination, and azithromycin in patients with pharyngitis in India: a real-world retrospective study from electronic medical records (PHARYSPOR)

**DOI:** 10.3389/fmed.2026.1743022

**Published:** 2026-07-09

**Authors:** Subramanyam Sarof, Nikita Kariappa Somana, Ravindra Channagiri S, Ramesh Chinthalapalli Venkataram, Jyothi Sharma R, Sabhapathi SV, Ramakrishnaiah Peddi, Sadanandam K, Nitin Rai Vohra, Lokesh Kumar Bhama, Mahendrakumar Shah, Vijay Chile, Ameeta Gunderia, Farad Momin, Anit Banerjee, Saumitra Kumar, Sandeep Rungta, Ashik Ikbal, Sunil Singhvi, Annamalai Natarajan, Monjori Mitra, Vijay Kadam, Shruti Dharmadhikari, Chintan Khandhedia, Gaurav Puppalwar, Neeraj Markandeywar, Amey Mane, Suyog Mehta

**Affiliations:** 1 Subramanyam Day Care Center, Bengaluru, India; 2 Aveksha Hospital, Bengaluru, India; 3 Ravi Clinic, Bengaluru, India; 4 Ramakrishna Hospital, Bengaluru, India; 5 Ramamurthy Nagar, Bengaluru, India; 6 Sri Ranganatha Clinic and Day Care Center, Bengaluru, India; 7 Langerhouse, Hyderabad, India; 8 Sadanand Nursing Home, Hyderabad, India; 9 Vohra ENT Care Center, Hyderabad, India; 10 Lotus Healthcare, Thane East, India; 11 Vinit Clinic, Mumbai, India; 12 Department of Internal Medicine, Aditya Nursing Home, Mumbai, India; 13 Nirmal Clinic, Mumbai, India; 14 Waris Nursing Home, Thane, India; 15 Department of Medicine, Sterling Hospital, Kolkata, India; 16 Jawaharlal Nehru Memorial Hospital, Kalyani, India; 17 Department of Internal Medicine and Cardiology, Manipal Hospital, Kolkata, India; 18 Department of Internal Medicine, R G Kar Medical College and Hospital, Kolkata, India; 19 Sri Singhvi Health Center, Chennai, India; 20 Apollo Clinic, Chennai, India; 21 Institute of Child Health, Kolkata, India; 22 Sun Pharma Laboratories Limited, Mumbai, India

**Keywords:** amoxicillin-potassium clavulanate combination, antimicrobial resistance (AMR), azithromycin, cephalexin, clavulanic acid, electronic medical records, pharyngitis, treatment outcome

## Abstract

**Background:**

Acute pharyngitis is a common condition in outpatient departments. Increasing antimicrobial resistance (AMR) necessitates careful prescribing practices. A new fixed-dose combination (FDC) of cephalexin-clavulanate has been approved in India for bacterial infections. This real-world evidence (RWE) study assessed the effectiveness and tolerability of cephalexin-CV compared to co-amoxiclav FDC and azithromycin in adult patients with acute bacterial pharyngitis.

**Methods:**

This retrospective, multicenter, comparative observational study (CTRI/2023/08/056474; Registered on: 14 Aug 2023) analyzed aggregated and anonymized electronic medical records (EMRs) of adults with clinically suspected acute bacterial pharyngitis who received study medications between November 2022 and March 2023. Patients with incomplete records, hospitalizations, or requiring parenteral antibiotics were excluded. Primary outcomes included the proportion of patients achieving clinical cure, i.e., complete resolution of symptoms (fever, sore throat, throat pain/difficulty during swallowing, inflammation of tonsils, alone or in combination) by Day 10 from treatment initiation. Secondary outcomes included time to clinical improvement (partial symptomatic resolution at Days 2, 3, 5, and 7 from treatment initiation), non-steroidal anti-inflammatory drug (NSAID) requirements, addition or change in antibiotics, and reported adverse events (AEs).

**Results:**

The study analyzed 752 patient data treated with cephalexin-CV (*n* = 285), co-amoxiclav (*n* = 245), and azithromycin (*n* = 222) as per the eligibility criteria. The overall clinical cure rates achieved by Day 10, clinical improvement at Day 7, and the mean time to improvement were similar across all treatment groups. However, cephalexin-CV demonstrated early clinical improvement by Day 2 [27 vs. 18% for co-amoxiclav (*p* = 0.035) and 15% for azithromycin (*p* = 0.003)] and Day 3 [58 vs. 47% (*p* = 0.022) and 48% (*p* = 0.071), respectively]. The need for NSAIDs was similar for the cephalexin CV and co-amoxiclav groups at 46% each, but was significantly higher at 57% in the azithromycin group (*p* = 0.020). AEs were minimal across all groups, including mild gastritis in the cephalexin CV group and cough, rash, and joint pain in the azithromycin group.

**Conclusion:**

This first-of-its-kind EMR-based RWE study did not demonstrate any statistically significant differences in overall clinical outcomes or tolerability among patients with pharyngitis treated with cephalexin-CV, co-amoxiclav, or azithromycin. However, cephalexin-CV was associated with earlier symptom improvement and lower NSAID use, making it a promising option for the treatment of pharyngitis.

**Key points**

- This study showed that the overall outcomes in treating pharyngitis in real-world practice using a new antibiotic, cephalexin-CV, were similar to common antibiotics like co-amoxiclav and azithromycin.
- Patients prescribed cephalexin-CV showed clinical improvement earlier compared to those prescribed co-amoxiclav and azithromycin.
- There was lower requirement for NSAIDs in patients consuming cephalexin-CV compared to those consuming azithromycin.
- Cephalexin-CV, offering early symptomatic relief and reduced requirement for NSAIDs, could be a useful option for doctors to consider when treating pharyngitis.

## 1 Introduction

Upper respiratory tract infections (URTIs) represent a significant global health burden. According to the Global Burden of Disease Study 2021, approximately 12.8 billion new episodes of URTIs were recorded globally across all ages and genders (1). The burden of respiratory infections in India is considerable. In 2019, the National Health Portal of India reported that URTIs represented 69% of all reported cases of infectious diseases in the country (2).

Bacterial pharyngitis accounts for approximately 5%−15% of URTIs in adults (3). *Streptococcus pyogenes* or group A beta-hemolytic streptococcus (GABHS) is the primary causative pathogen (4), responsible for an estimated 616 million cases of acute bacterial pharyngitis annually worldwide (5) with high requirements for medical attention in tertiary care settings (6). It typically presents with the sudden onset of sore throat, pain during swallowing, fever, headache, abdominal pain, nausea, vomiting (especially in children), tonsillar exudates, and tender cervical lymphadenopathy (7).

Bacterial pharyngitis can significantly reduce the health-related quality of life (HRQoL) of patients (8). On average, a single episode of GABHS pharyngitis in adults can lead to missing 1.8 days of work. Recurrent episodes of bacterial pharyngitis can cause significant psychological distress, particularly manifesting as anxiety in both children and their parents (9).

**Figure 1:**
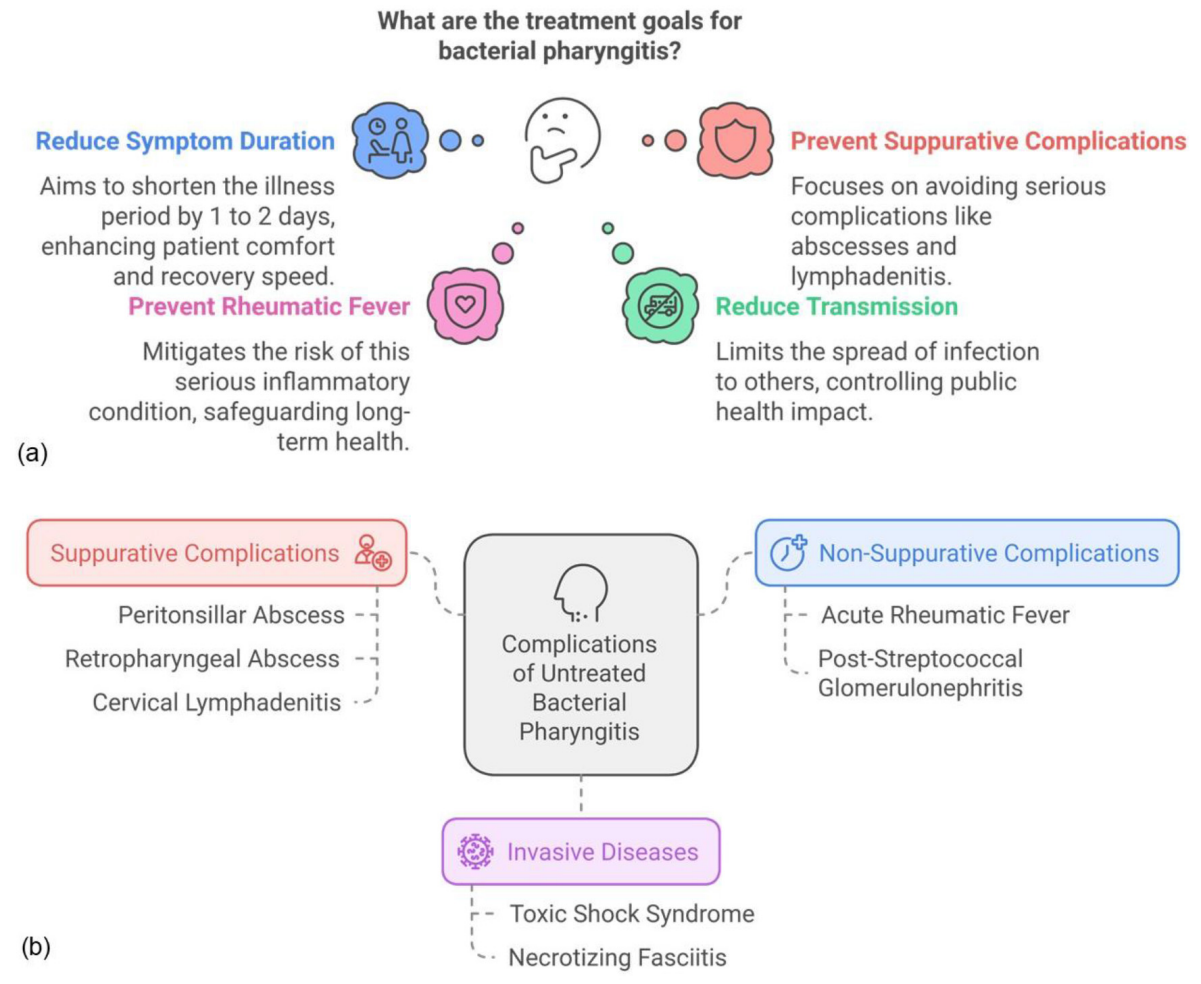
**(a)** Treatment goals for bacterial pharyngitis, **(b)** Complications of untreated bacterial pharyngitis. The image, infographics, and design are generated using AI from the text in the manuscript (source: https://www.napkin.ai/).

The primary goals of bacterial pharyngitis treatment are symptomatic improvement, preventing complications (including peritonsillar abscess, rheumatic fever, and glomerulonephritis), and limiting disease transmission, (10, 11), as untreated cases lead to severe outcomes like peritonsillar and retropharyngeal abscess, cervical lymphadenitis, acute rheumatic fever, post-streptococcal glomerulonephritis, toxic shock syndrome, and necrotizing fasciitis (Figure 1) (9).

According to practice guidelines for diagnosis and management of group A streptococcal pharyngitis by Bisno et al., antibiotic therapy is the mainstay of treatment for bacterial pharyngitis (4, 12–14). Accurate diagnosis is crucial to avoid unnecessary antibiotic use and to reduce the chances of antimicrobial resistance (AMR) (15, 16).

Some of the typical bacterial flora found in the pharynx and tonsils generate beta-lactamase, which protects GABHS from the effects of penicillin (17). Antimicrobials targeting beta-lactamase-producing organisms have been demonstrated to achieve higher rates of GABHS eradication compared to penicillin (18). To address the problem of beta-lactamase production and prevent AMR, beta-lactamase inhibitors are administered alongside beta-lactam antimicrobials (19). Cephalexin-clavulanate (Cephalexin-CV), a first-of-its-kind fixed-dose combination (FDC), represents a combination of a beta-lactam and a beta-lactamase inhibitor (BL/BLI) that provides a synergistic effect to improve the antimicrobial activity of cephalexin against beta-lactamase-producing strains (20, 21). Data on the prevalence and management of pharyngitis in Indian adults are limited, as most studies have concentrated on the pediatric population. Despite multiple antibiotics being available to manage bacterial infections like pharyngitis, there is a lack of data comparing commonly prescribed antibiotics in the country. Moreover, no recent research has compared the response of adult Indian patients with bacterial pharyngitis to the cephalexin-CV FDC and other guideline-recommended antibiotics such as co-amoxiclav and azithromycin in real-world settings. We aimed to evaluate the effectiveness and tolerability of cephalexin-CV FDC compared to co-amoxiclav FDC and azithromycin in adults with signs and symptoms suggestive of bacterial pharyngitis in this real-world retrospective study from electronic medical records (PHARYSPOR).

## 2 Methods

This was a real-world, retrospective, multicentric, observational study (CTRI/2023/08/056474) using aggregated and anonymized data retrieved from electronic medical records (EMRs) of patients treated for acute pharyngitis between November 2022 and March 2023. EMRs were retrieved from 20 participating centers across India (located in and around Bangalore, Chennai, Hyderabad, Kolkata, and Mumbai). Data was retrieved for up to 10 days after treatment initiation with any of the following medications: cephalexin-CV [FDC of 375/750 mg cephalexin extended release and potassium clavulanate diluted IP (equivalent to clavulanic acid 125 mg)], FDC of amoxicillin (500 mg) and clavulanic acid (125 mg; co-amoxiclav), or azithromycin (500 mg).

The study was conducted following the principles of the Declaration of Helsinki (22) and the International Council for Harmonization Good Clinical Practice guidelines (23). The study conformed to the Indian Council for Medical Research (ICMR) and Indian Good Clinical Practice (GCP) guidelines for clinical research. The study involved extracting aggregated and anonymized data from the EMR database, and permission for an Informed Consent Form (ICF) waiver was obtained before study initiation from the Institutional Ethics Committee (IEC) in accordance with the ICMR-National Ethical Guidelines for Biomedical and Health Research Involving Human Participants, 2017 (24). Data from EMRs were retrieved only after study approval was obtained from the IEC and the study was registered on CTRI. Data quality was checked in terms of accuracy, reliability, and consistency. Extracted data was cross-verified with the EMRs.

The study included male and female patients (age ≥18 years) diagnosed with clinically suspected acute pharyngitis and who were prescribed oral cephalexin-CV, co-amoxiclav, or azithromycin by the treating physicians. Clinical diagnosis was based on the symptoms suggestive of bacterial pharyngitis, alone or in combination, including fever, sore throat, throat pain, dysphagia, or tonsillar inflammation. Patients with incomplete medical records, hospitalizations, and the need for intravenous antibiotics were excluded.

The primary endpoint was the proportion of patients achieving clinical cure (complete resolution of infection-related clinical signs and symptoms, i.e., fever, sore throat, throat pain/difficulty during swallowing, inflammation of tonsils) till 10 days from treatment initiation. Secondary endpoints included the proportion of patients achieving clinical improvement (partial resolution of infection-related clinical signs and symptoms, i.e., fever, sore throat, throat pain/difficulty during swallowing, inflammation of tonsils) at Days 2, 3, 5, and 7 from treatment initiation, time to clinical improvement, the proportion of patients requiring addition of non-steroidal anti-inflammatory drugs (NSAIDs), proportion of patients requiring addition/change in antibiotics, and proportion of patients with adverse events (AEs).

Comparing the clinical cure rate of cephalexin-CV (94%) to that of azithromycin (99%) (25), the total sample size for the study was estimated to be 498 with 80% power and a 5% level of significance. Considering Bonferroni adjustment for comparison across three groups, the total sample size computed was 633 with a 2.5% level of significance. Anticipating 15% missing data, EMRs of at least 750 patients were required at baseline (i.e., the observations recorded before the start of antibiotic treatment in patients).

Statistical methods were based on the International Council for Harmonization (ICH) E9 document ‘Statistical Principles for Clinical Trials'; all analyses were performed using SPSS version 28.0.1.1 (IBM Corp., Armonk, NY, USA) and R 4.5.1. Hypothesis testing was conducted at a 2.5% (two-sided) level of significance; *p* < 0.025 was considered statistically significant. One-way Analysis of Variance (ANOVA) and *post hoc* test (with Bonferroni correction) were used for comparing continuous variables, and the Chi-square test was used for comparing categorical variables between groups.

Age, gender, temperature, treatment duration, concomitant medications (NSAID/Non-NSAID), baseline symptom load, zone were considered as covariates. Missing data in covariates (Supplementary Table S1) were addressed using multiple imputation by chained equations (MICE) implemented in the *mice* package. Continuous variables (age and temperature) were imputed using predictive mean matching, while categorical variables (gender) were imputed using classification and regression trees. Analyses were performed across multiple imputed datasets, and final estimates were combined using Rubin's rules to account for within- and between-imputation variability.

For clinical cure, crude odds ratios (ORs) were estimated using standard logistic regression. For clinical improvement, crude ORs were derived using generalized estimating equations (GEE) to account for repeated measures across visits. To adjust for potential confounding in treatment allocation, propensity scores were estimated using multinomial logistic regression including baseline covariates (age, temperature, gender, NSAID use, treatment duration, Zone, and symptom load). Stabilized inverse probability of treatment weights (IPTW) were constructed from these propensity scores, and extreme weights were truncated at the 5th and 95th percentiles to improve model stability. Variables with high cardinality, such as prescriber, were excluded from the propensity score model to avoid instability.

Adjusted treatment effects for clinical cure were estimated using weighted logistic regression within a survey design framework to account for IPTW. For longitudinal clinical improvement outcomes, weighted GEE models with a Poisson distribution and log link were fitted to account for repeated observations across visits. Treatment effects, including drug-by-visit interactions, were estimated using marginal contrasts from the fitted models. Results are reported as adjusted odds ratios (ORs) with 95% confidence intervals (CIs). Finally, all estimates were pooled across imputed datasets using Rubin's rules to obtain overall inference.

## 3 Results

**Figure 2:**
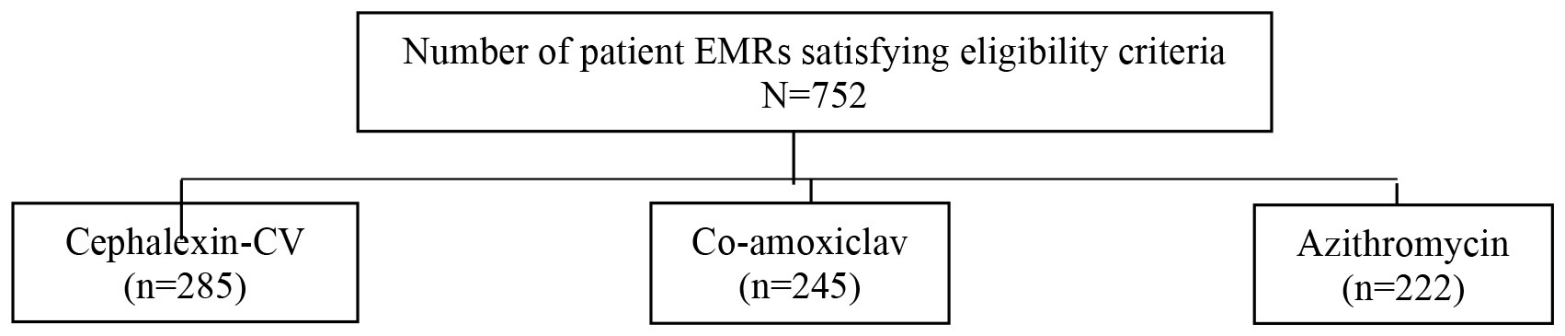
Screened and analyzed patient EMRs.

Aggregated and anonymized EMR data from 752 patients were included in the study. Of these, 285 (37.90%) patients were prescribed cephalexin-CV [375 mg cephalexin, 149 (52.28%); 750 mg cephalexin, 136 (47.72%)], 245 (32.58%) were prescribed co-amoxiclav, and 222 (29.52%) were prescribed azithromycin (Figure 2). The mean ± SD age of the study population was 43.08 ± 15.35 years in cephalexin-CV, 38.52 ± 13.61 years in co-amoxiclav, and 38.61 ± 13.32 years in azithromycin. Baseline characteristics, including weight, height, and gender, were similar among the three groups. The mean ± SD temperature of patients was 100.98 ± 1.17°F, 100.18 ± 1.32°F, and 100.78 ± 0.89°F in the cephalexin-CV, co-amoxiclav, and azithromycin groups, respectively. Systolic Blood Pressure (SBP), Diastolic Blood Pressure (DBP), pulse rate, and respiratory rate were also similar among the three groups. The symptom load in the cephalexin-CV group was higher compared to the co-amoxiclav and azithromycin groups, with 93.68% (267/285) reporting fever, 51.58% (147/285) reporting sore throat, 36.49% (104/285) with throat pain/difficulty during swallowing, and 24.91% (71/285) with inflamed tonsils (Table 1).

**Table 1:** Baseline characteristics of patients.

Parameter	Total (*N* = 752)	Cephalexin-CV (*n* = 285)	Co-amoxiclav (*n* = 245)	Azithromycin (*n* = 222)
Age (years), mean ± SD	40.37 ± 14.40	43.08 ± 15.35	38.52 ± 13.61	38.61 ± 13.32
Weight (kg), mean ± SD	68.68 ± 8.13	68.06 ± 8.26	67.24 ± 8.79	70.79 ± 6.98
Height (cm), mean ± SD	163.13 ± 6.49	163.14 ± 6.59	162.37 ± 6.71	163.67 ± 6.21
Gender				
Male (%)	360 (47.87%)	147 (51.58%)	109 (44.49%)	104 (46.85%)
Female (%)	352 (46.81%)	130 (45.61%)	108 (44.08%)	114 (51.35%)
Data not available (%)	40 (5.32%)	8 (2.81%)	28 (11.43%)	4 (1.80%)
Vital signs, mean ±SD				
SBP (mmHg)	123.24 ± 12.04	123.10 ± 10.44	124.26 ± 14.57	122.82 ± 11.87
DBP (mmHg)	81.42 ± 12.04	81.31 ± 6.68	82.78 ± 9.07	80.79 ± 7.42
Pulse rate (per minute)	94.46 ± 12.43	94.81 ± 12.17	94.76 ± 11.14	93.99 ± 13.37
Respiratory rate (per minute)	26.82 ± 8.37	22.33 ± 6.98	28.24 ± 8.54	26.95 ± 8.50
Temperature (°F)	100.66 ± 1.20	100.98 ± 1.17	100.18 ± 1.32	100.78 ± 0.89
Clinical symptoms, *n* (%)				
Fever	676 (89.89%)	267 (93.68%)	195 (79.59%)	214 (96.40%)
Sore throat	381 (50.66%)	147 (51.58%)	129 (52.65%)	105 (47.30%)
Throat pain/difficulty in swallowing	285 (37.90%)	104 (36.49%)	76 (31.02%)	105 (47.30%)
Inflamed tonsils	141 (18.75%)	71 (24.91%)	39 (15.92%)	31 (13.96%)
Diagnosis, *n* (%)				
Acute pharyngitis	752 (100.00%)	285 (100.00%)	245 (100.00%)	222 (100.00%)
a) with tonsillitis	69 (9.17%)	34 (11.93%)	21 (8.57%)	14 (6.31%)
b) with bronchitis	4 (0.53%)	1 (0.35%)	2 (0.82%)	1 (0.45%)

Most patients (55.08%) in cephalexin-CV were prescribed the antibiotic for 7 days, while most patients in the other two groups were prescribed antibiotics for 5 days (co-amoxiclav, 60.40%; azithromycin, 95.50%) by the treating physicians. Among concomitant medications consumed by patients, NSAID usage was the highest (19.95%), followed by the usage of expectorants (16.09%), antiallergic and leukotriene receptor antagonists (13.03%), and others (Table 2).

**Table 2:** Comorbidities, treatment details, and concomitant medication use in patients.

Parameters	Total (*N* = 752)	Cephalexin-CV (*n* = 285)	Co-amoxiclav (*n* = 245)	Azithromycin (*n* = 222)
Comorbidity, *n* (%)				
Hypertension	17 (2.26%)	7 (2.46%)	3 (1.22%)	7 (3.15%)
Dyslipidemia	6 (0.8%)	2 (0.7%)	0 (0%)	4 (1.8%)
Asthma	6 (0.8%)	3 (1.05%)	1 (0.41%)	2 (0.9%)
Type 2 diabetes mellitus	5 (0.66%)	3 (1.05%)	0 (0%)	2 (0.9%)
Parasitic worm infection	2 (0.27%)	1 (0.35%)	1 (0.41%)	0 (0%)
Insomnia	2 (0.27%)	1 (0.35%)	0 (0%)	1 (0.45%)
Hypothyroidism	2 (0.27%)	2 (0.7%)	0 (0%)	0 (0%)
Hypocalcemia	2 (0.27%)	2 (0.7%)	0 (0%)	0 (0%)
Chickenpox	1 (0.13%)	1 (0.35%)	0 (0%)	0 (0%)
Prostate enlargement	1 (0.13%)	0 (0%)	1 (0.41%)	0 (0%)
Frequency of administration, *n* (%)				
OD		0 (0%)	0 (0%)	132 (59.46%)
BID		282 (98.95%)	227 (92.65%)	85 (38.29%)
TID		3 (1.05%)	18 (7.35%)	5 (2.25%)
Treatment duration, *n* (%)				
5 days		121 (42.45%)	148 (60.40%)	212 (95.50%)
7 days		157 (55.08%)	65 (26.53%)	7 (3.15%)
10 days		7 (2.45%)	32 (13.06%)	3 (1.35%)
Concomitant medication, *n* (%)				
NSAID	150 (19.95%)	50 (17.54%)	70 (28.57%)	30 (13.51%)
Expectorant	121 (16.09%)	49 (17.19%)	42 (17.14%)	30 (13.51%)
Antiallergic and leukotriene receptor antagonist	98 (13.03%)	36 (12.63%)	33 (13.47%)	29 (13.06%)
Vitamin	50 (6.65%)	16 (5.61%)	13 (5.31%)	21 (9.46%)
Anti-microbial/antiseptic	20 (2.66%)	10 (3.51%)	7 (2.86%)	3 (1.35%)
Proton pump inhibitor (PPI)	17 (2.26%)	10 (3.51%)	6 (2.45%)	1 (0.45%)
Antiallergic	14 (1.86%)	7 (2.46%)	6 (2.45%)	1 (0.45%)
Bronchodilator	13 (1.73%)	2 (0.7%)	10 (4.08%)	1 (0.45%)
Angiotensin receptor blockers (ARBs)	10 (1.33%)	3 (1.05%)	3 (1.22%)	4 (1.8%)
Corticosteroids	6 (0.8%)	3 (1.05%)	1 (0.41%)	2 (0.9%)
Proteolytic enzymes	5 (0.66%)	2 (0.7%)	3 (1.22%)	0 (0%)
Statins	3 (0.4%)	1 (0.35%)	0 (0%)	2 (0.9%)

OD, once daily; BD, twice daily (Bis in Die); TID, three times daily (Ter in Die); NSAID, non-steroidal anti-inflammatory drug.

**Figure 3:**
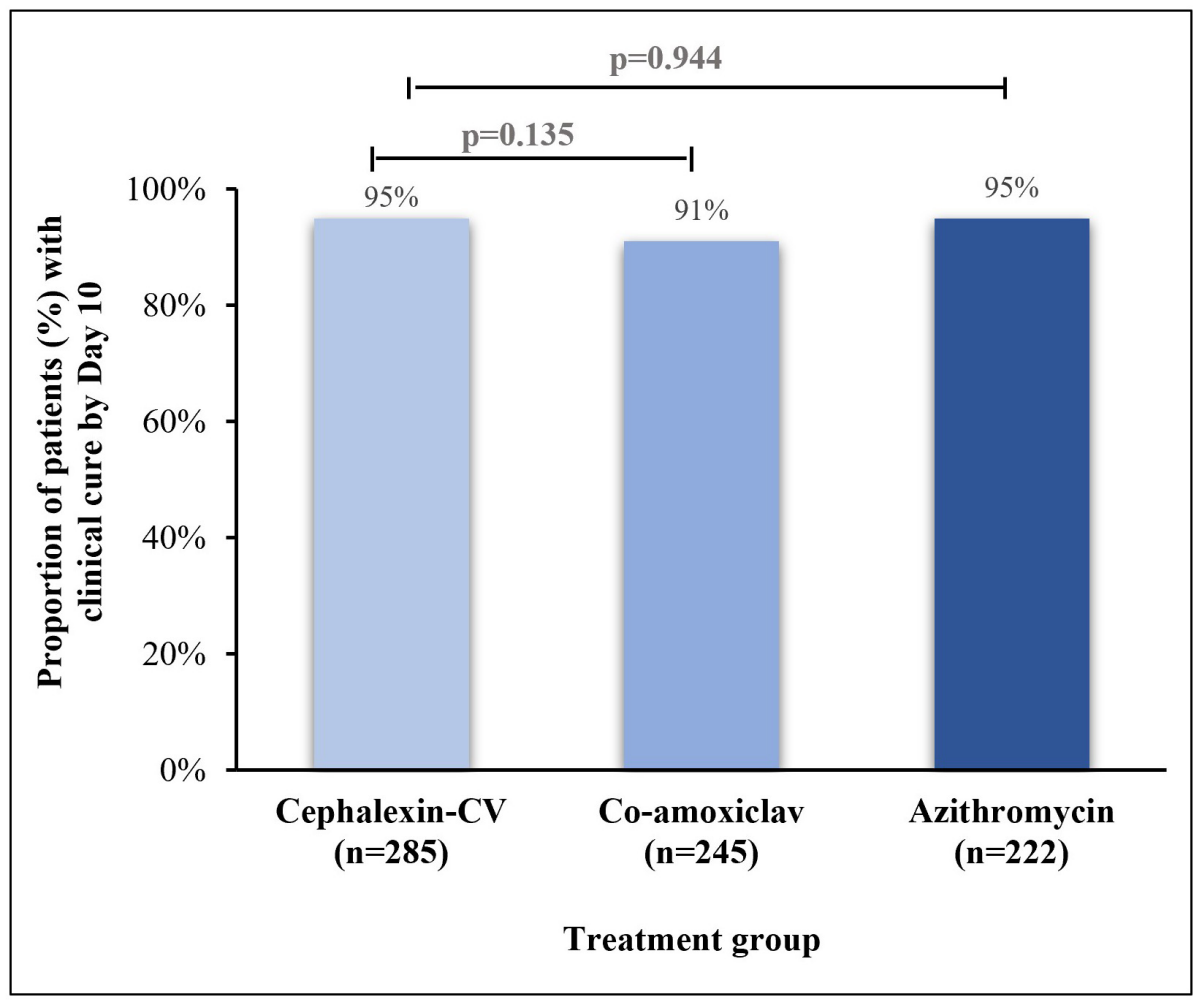
Proportion of patients achieving clinical cure by day 10 post-treatment initiation.

The proportion of patients achieving clinical cure by Day 10 from treatment initiation with cephalexin-CV (95%) did not differ significantly from the corresponding proportions obtained with co-amoxiclav (91%; OR: 0.59, 95% CI: 0.30–1.18, *p* = 0.135) and azithromycin (95%; OR: 0.97, 95% CI: 0.45–2.12, *p* = 0.944; (Table 3 and Figure 3). Similar observations were made using adjusted OR (Table 2).

**Table 3:** Clinical cure and clinical improvement: comparison among treatment groups.

Treatment group	Number of patients (%)	Crude OR [95% CI]	*p*-value	Adjusted OR [95% CI]	*p*-value
Clinical cure by day 10					
Cephalexin-CV (*n* = 285)	270 (95%)	1 (reference)	—	1 (reference)	—
Co-amoxiclav (*n* = 245)	224 (91%)	0.59 [0.30, 1.18]	0.135	0.74 [0.33, 1.65]	0.460
Azithromycin (*n* = 222)	210 (95%)	0.97 [0.45, 2.12]	0.944	1.09 [0.48, 2.49]	0.840
Clinical improvement by:					
Day 2					
Cephalexin-CV (*n* = 285)	76 (27%)	1 (Reference)	—	1 (Reference)	—
Co-amoxiclav (*n* = 245)	44 (18%)	0.60 [0.38, 0.97]	0.035	0.68 [0.47, 1.00]	0.052
Azithromycin (*n* = 222)	33 (15%)	0.48 [0.29, 0.80]	**0.003** ^**^	0.68 [0.44, 1.06]	0.090
Day 3					
Cephalexin-CV (*n* = 285)	164 (58%)	1 (Reference)	—	1 (Reference)	—
Co-amoxiclav (*n* = 245)	114 (47%)	0.64 [0.43, 0.95]	**0.022** ^*^	0.84 [0.70, 1.02]	0.082
Azithromycin (*n* = 222)	107 (48%)	0.69 [0.46, 1.03]	0.071	1.02 [0.86, 1.21]	0.824
Day 5					
Cephalexin-CV (*n* = 285)	223 (78%)	1 (Reference)	—	1 (Reference)	—
Co-amoxiclav (*n* = 245)	213 (87%)	1.85 [1.09, 3.14]	**0.019** ^*^	1.00 [0.92, 1.09]	0.961
Azithromycin (*n* = 222)	188 (85%)	1.54 [0.91, 2.60]	0.123	1.10 [1.02, 1.19]	**0.014** ^*^
Day 7					
Cephalexin-CV (*n* = 285)	263 (92%)	1 (Reference)	—	1 (Reference)	—
Co-amoxiclav (*n* = 245)	231 (94%)	1.38 [0.63, 3.03]	0.561	1.01 [0.94, 1.09]	0.727
Azithromycin (*n* = 222)	215 (97%)	2.57 [0.96, 6.90]	0.063	1.08 [1.01, 1.15]	0.026

^*^p < 0.025; ^**^p < 0.01.

**Figure 4:**
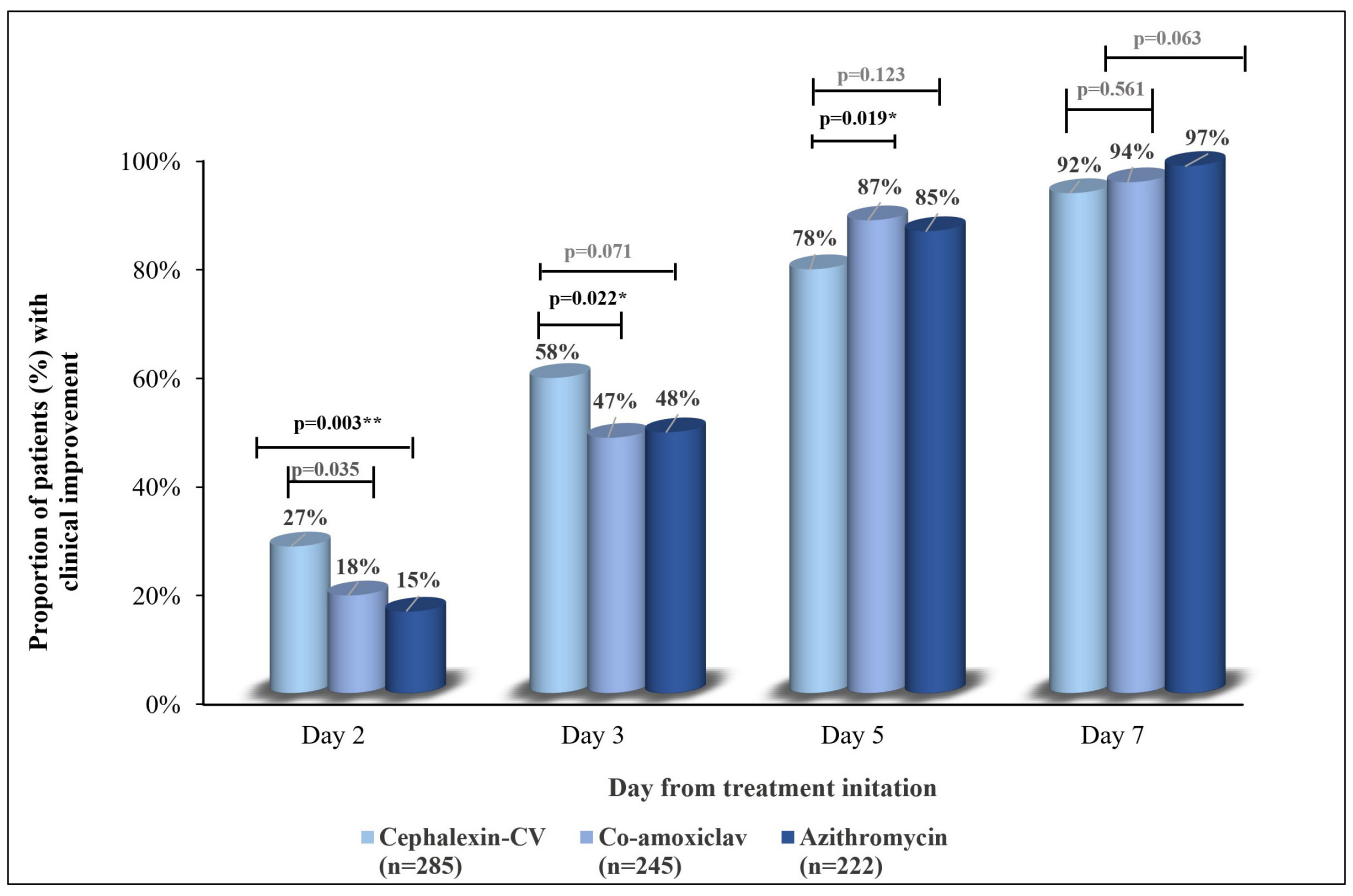
Proportion of patients achieving clinical improvement by Day 2, 3, 5, and 7 post-treatment initiation. ^*^p < 0.025, ^**^p < 0.01.

The proportion of patients with clinical improvement by Day 2 upon treatment with cephalexin-CV (27%) was significantly higher than that with azithromycin (15%; OR: 0.48, 95% CI: 0.29–0.80, *p* = 0.003), while improvements at Days 3, 5, and 7 were not significantly different between the two treatments (Table 3 and Figure 4).

Likewise, the proportion of patients with clinical improvement upon cephalexin-CV treatment at Day 3 (58%) was significantly higher than that with co-amoxiclav (47%; OR: 0.64, 95% CI: 0.43–0.95, *p* = 0.022). By Day 5, however, co-amoxiclav showed significantly better improvement compared to cephalexin-CV (87 vs. 78%; OR: 1.85, 95% CI: 1.09–3.14, *p* = 0.019). The two treatment arms showed statistically similar improvement at Days 2 and 7 (Table 3 and Figure 4).

**Figure 5:**
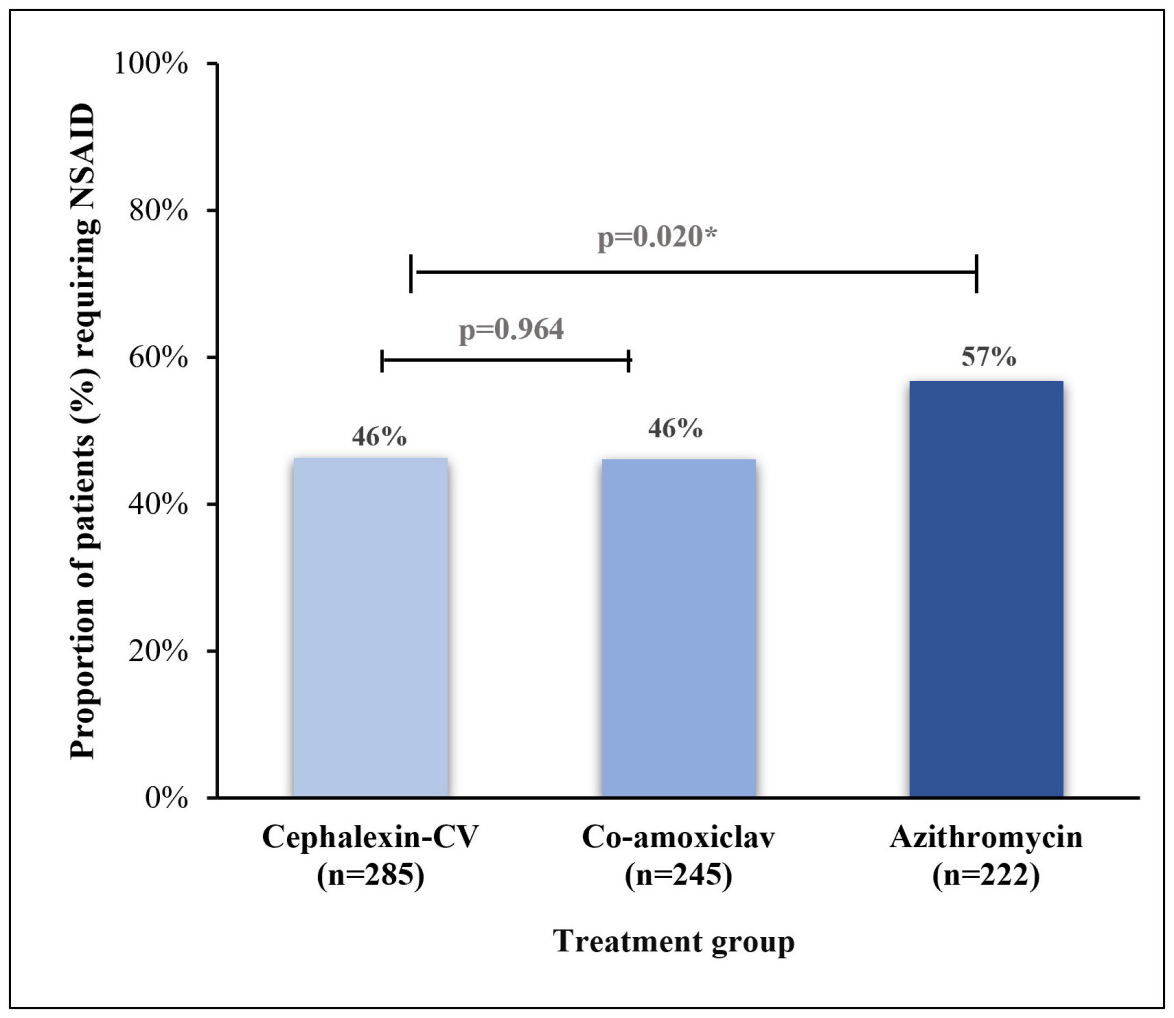
Proportion of patients requiring the addition of an NSAID. ^*^p < 0.025.

Compared to the cephalexin-CV group, the proportion of patients requiring additional NSAIDs was similar in the co-amoxiclav group (46 vs. 46%; *p* = 0.964) and significantly higher in the azithromycin group (46% vs. 57%; *p* = 0.020; Table 4 and Figure 5).

**Table 4:** Overall NSAID requirement among treatment groups.

Antibiotic groups (*n* = 752)	Number of Patients with NSAID requirement (%)	Difference with cephalexin-CV	
		[95% CI]	*p*-value
NSAID requirement			
Cephalexin-CV (*n* = 285)	132 (46%)	—	—
Co-amoxiclav (*n* = 245)	113 (46%)	[−8.32%, 8.71%]	0.964
Azithromycin (*n* = 222)	126 (57%)	[−19.16%, −1.72%]	**0.020** ^*^

^*^p < 0.025.

The mean ± SD time to clinical improvement was 4.01 ± 2.22 days in the cephalexin-CV group, 3.91 ± 1.87 days in the co-amoxiclav group, and 3.81 ± 1.48 days in the azithromycin group. The median times to clinical improvement were 3, 4, and 4 days in the cephalexin-CV, co-amoxiclav, and azithromycin groups, respectively. Three patients in the azithromycin group did not show clinical improvement and, therefore, were excluded from this analysis (Table 5).

**Table 5:** Time to clinical improvement: comparison among treatment groups.

Antibiotic groups (*N* = 752)	Time to clinical improvement (days)		*p*-value for difference with Cephalexin-CV
	Mean ±SD	Median (min, max)	
Cephalexin-CV (*n* = 285)	4.01 ± 2.22	3 (2,10)	—
Co-amoxiclav (*n* = 245)	3.91 ± 1.87	4 (2,10)	0.999
Azithromycin (*n* = 222)	3.81 ± 1.48	4 (2,10)	0.726

Three patients in the azithromycin group did not show clinical improvement and were excluded from this analysis.

In the azithromycin group, fever did not resolve, and cough was evident on Day 7 in one patient. Hence, the antibiotic was changed to co-amoxiclav. No change in prescribed antibiotics was required in the other groups.

One patient (0.35%) in the cephalexin-CV group reported mild gastritis. In azithromycin, two patients (0.90%) reported AEs; one reported cough, and another reported skin rash and joint pain. No AE was reported in the co-amoxiclav group. None of the reported AEs were severe.

## 4 Discussion

This retrospective, multicentric, and observational PHARYSPOR study analyzed 752 EMRs of patients across India with clinically suspected acute bacterial pharyngitis who were prescribed cephalexin-CV FDC, co-amoxiclav FDC, or azithromycin. The proportion of patients achieving clinical cure after completion of individual therapy by Day 10 using cephalexin-CV was similar to those using co-amoxiclav FDC or azithromycin. This proportion after completion of individual therapy by Day 10 remained similar after adjusting for age, temperature, gender, use of concomitant medications, and treatment duration. The proportion of patients with partial resolution of symptoms, such as fever, sore throat, throat pain/difficulty during swallowing, and inflamed tonsils was significantly higher with cephalexin-CV compared to azithromycin at Day 2 of treatment initiation, while it was significantly higher compared to co-amoxiclav at Day 3 of treatment initiation. The overall time to clinical improvement was not significantly different across the three groups. Additionally, compared to the cephalexin-CV group, more patients using azithromycin needed paracetamol and other NSAIDs for symptom relief. In terms of AEs, one patient in the cephalexin-CV group reported mild gastritis, and two patients in the azithromycin group had mild AEs. None reported serious AEs.

These results align with prior studies suggesting the efficacy of cephalexin-CV in treating streptococcal pharyngitis and its advantages of rapid absorption and broad-spectrum efficacy (26, 27). Banerjee et al. reported that cephalexin-CV provides faster symptom relief, which can significantly impact QoL and reduce absenteeism in patients with dental infections. The retrospective analysis of real-world EMR data of 355 patients with dental infection showed that cephalexin-CV produced outcomes similar to cefuroxime and co-amoxiclav, with faster clinical improvement and better resolution of certain symptoms, concordant with the findings of the current study (28). A review article by Cots et al. (10) that provides recommendations for treatment of acute pharyngitis in adults states that the antibiotics of choice include penicillin and amoxicillin. Our findings align with the study, as we did not observe significant clinical cure or improvement with co-amoxiclav FDC in comparison to cephalexin-CV. Similar to this article, we also did not find significant clinical cure or improvement with azithromycin compared to cephalexin-CV. A recent study that assessed the methodological quality of the available clinical guidelines for laryngitis and pharyngitis found a lack of uniformity in both pharmacological and non-pharmacological recommendations (29). The use of penicillin, erythromycin, amoxicillin, azithromycin, and clarithromycin was recommended in 75% of the guidelines that provided pharmacological measures. In the absence of uniform practice guidelines for the treatment of acute pharyngitis and based on our findings, further large-scale studies for using cephalexin-CV in acute pharyngitis management are needed. A systematic review and clinical practice recommendation for treatment of recurrent acute tonsillitis by Guntinas-Lichius et al. (30) states that antimicrobial therapy has no proven benefit in acute pharyngitis not caused by GABHS. Similar to our study, antibiotics remain the mainstay treatment for acute bacterial pharyngitis, and in the absence of Indian guidelines for rational therapy, newer combinations like cephalexin-CV need to be evaluated at the population level.

This is the first real-world study that offers valuable insights into the comparative effectiveness and tolerability of cephalexin-CV, co-amoxiclav, and azithromycin. The rise in beta-lactamase production by normal flora can lead to AMR, affecting the management of bacterial pharyngitis. This can undermine the effectiveness of penicillin, as beta-lactamases can inactivate the beta-lactam antibiotics (12, 31, 32). To address this, BLIs like clavulanic acid may be necessary to maintain the antibiotic's antibacterial activity and prevent resistance development in non-beta-lactamase-producing pathogens (12, 32). The study outcomes are deemed to guide healthcare practitioners in making informed clinical decisions for the treatment and management of bacterial pharyngitis, assisting them in optimizing treatment strategies and minimizing the risk of antibiotic resistance.

Evidence-based research on antibiotic efficacy in bacterial pharyngitis proved essential for clinical decisions, particularly in cases unresponsive to amoxicillin or with recurrent infections. The Infectious Diseases Society of America (IDSA) guidelines list cephalexin as a strong recommendation with high-quality evidence for patients with penicillin allergy (4, 33). Cephalexin has emerged as an effective first- and second-line alternative to penicillin and is generally well-tolerated, with adverse effects occurring relatively infrequently (34). Results from our study demonstrate the effectiveness and tolerability of cephalexin-CV; however, the findings do not justify modification of existing recommendations for first-line treatment. Although the Centor criteria guide in diagnosis and the appropriate use of antibiotics (35), limited real-world data restrict the comparison of the effectiveness and tolerability of cephalexin-CV with co-amoxiclav and azithromycin in managing bacterial pharyngitis. The Centor criteria guide is used by physicians for acute pharyngitis management, as bacterial culture and sensitivity-based results commonly yield false positives. Our study primarily used the Centor criteria for patient diagnosis; however, a small subset of participants with cough were included to ensure the data reflected a real-world patient population. A narrative review that evaluated diagnostic techniques, clinical guidelines, and antibiotic treatment for acute pharyngitis grouped the diagnostic techniques into clinical scoring systems, rapid antigen testing methods, throat culture, and nucleic acid amplification (36). According to this study, having been used as diagnostic tools since the 1980s, clinical scoring systems like the Centor and McIsaac criteria are considered valuable and time-tested. Rapid antigen detection, throat culture, and nucleic acid amplification tests are time-consuming, expensive, and often fail to distinguish between an active infection and carrier state.

This study had several limitations. First, this is a retrospective study, which relied on previously collected data from electronic health records, that may have missing or incomplete recorded information, which limits the assessment of long-term outcomes. There also exists a possibility that adverse drug reactions were not adequately or completely recorded in the EMRs. Second, the baseline values for signs and symptoms did not match across all groups. We tried addressing this by acknowledging that the data derived from EMRs reflects real-world clinical scenarios. Third, the lack of randomization and comparison with a control group could have led to selection and confounding bias. We tried minimizing selection bias as our participants were not chosen to be in one of the three groups based on their previous health status. We acknowledge that confounding bias is a major concern in studies without randomization; however, we have reported results after adjusting for several covariates. The unavailability of laboratory data may introduce misclassification bias. As clinicians typically diagnose pharyngitis based on the Centor criteria as per clinical signs and symptoms, we believe this minimizes the risk of misclassification. The study ultimately captured the real-world application of antibiotic therapy, revealing a lack of uniformity in the duration of antibiotic treatment based on various guidelines, expert opinions, and certain publications advocating for shorter treatment courses. This variation was further assessed through patient-reported outcomes via telephone consultations and evaluations by physicians. Given these findings, it may be particularly valuable to consider conducting a study focused on antibiotic treatment duration, both short- and long-term, in the context of rising antimicrobial resistance.

## 5 Conclusion

This study demonstrated that the effectiveness and tolerability of cephalexin-CV were not significantly different than co-amoxiclav and azithromycin. The early improvement of clinical signs and symptoms, along with lower requirement for NSAIDs, was significantly better with cephalexin-CV treatment compared to both co-amoxiclav and azithromycin. The findings may be considered for optimizing treatment strategies. Further randomized controlled trials and prospective studies across diverse demographics would strengthen these results and confirm the broader clinical utility of cephalexin CV.

## Data Availability

The raw data supporting the conclusions of this article will be made available by the authors, without undue reservation.
