# Supplementary material for: Effectiveness and tolerability of cephalexin and clavulanic acid fixed dose combination, amoxicillin and clavulanic acid fixed dose combination, and azithromycin in patients with pharyngitis in India: a real-world retrospective study from electronic medical records (PHARYSPOR)
Source: Front Med (Lausanne). 2026 Jul 9;13:1743022. doi: 10.3389/fmed.2026.1743022 (PMC13391552; doi:10.3389/fmed.2026.1743022)
Supplement: Supplementary file 1 [file Data_Sheet_1.pdf]

Supplementary material

Effectiveness and tolerability of cephalexin and clavulanic acid fixed dose combination, amoxicillin and clavulanic acid fixed dose combination, and azithromycin in patients with pharyngitis in India: a real-world retrospective study from electronic medical records (PHARYSPOR)

Short title: *Cephalexin-Clavulanic Acid in Pharyngitis*

In this real-world retrospective study from electronic medical records, a few data were missing data with respect to the following variables. The corresponding numbers are mentioned in the following table:

Supplementary Table S1: Number of missing values in covariates

	Number of missing values		
**Variables**	**Age**	**Gender**	**Temperature**
**Total**	76	40	4
**Cephalexin-CV**	15	8	3
**Co-amoxiclav**	42	28	0
**Azithromycin**	19	4	1
